# Erythrocyte Pyruvate Kinase Deficiency mutation identified in multiple breeds of domestic cats

**DOI:** 10.1186/1746-6148-8-207

**Published:** 2012-10-30

**Authors:** Robert A Grahn, Jennifer C Grahn, Maria CT Penedo, Chris R Helps, Leslie A Lyons

**Affiliations:** 1 Department of Population Health and Reproduction, School of Veterinary Medicine, University of California, Davis, CA, 95616, USA; 2 Veterinary Genetics Laboratory, School of Veterinary Medicine, University of California, Davis, CA, 95616, USA; 3 Molecular Diagnostic Unit, Langford Veterinary Services, University of Bristol, Langford, BS40 5DU, UK

**Keywords:** *PKLR*, Pyruvate kinase liver and RBC, Pyruvate kinase deficiency, PK deficiency, Feline, Cat, Anemia

## Abstract

**Background:**

Erythrocyte pyruvate kinase deficiency (PK deficiency) is an inherited hemolytic anemia that has been documented in the Abyssinian and Somali breeds as well as random bred domestic shorthair cats. The disease results from mutations in *PKLR*, the gene encoding the regulatory glycolytic enzyme pyruvate kinase (PK). Multiple isozymes are produced by tissue-specific differential processing of *PKLR* mRNA. Perturbation of PK decreases erythrocyte longevity resulting in anemia. Additional signs include: severe lethargy, weakness, weight loss, jaundice, and abdominal enlargement. In domestic cats, PK deficiency has an autosomal recessive mode of inheritance with high variability in onset and severity of clinical symptoms.

**Results:**

Sequence analysis of *PKLR* revealed an intron 5 single nucleotide polymorphism (SNP) at position 304 concordant with the disease phenotype in Abyssinian and Somali cats. Located 53 nucleotides upstream of the exon 6 splice site, cats with this SNP produce liver and blood processed mRNA with a 13 bp deletion at the 3’ end of exon 5. The frame-shift mutation creates a stop codon at amino acid position 248 in exon 6. The frequency of the intronic SNP in 14,179 American and European cats representing 38 breeds, 76 western random bred cats and 111 cats of unknown breed is 6.31% and 9.35% when restricted to the 15 groups carrying the concordant SNP.

**Conclusions:**

PK testing is recommended for Bengals, Egyptian Maus, La Perms, Maine Coon cats, Norwegian Forest cats, Savannahs, Siberians, and Singapuras, in addition to Abyssinians and Somalis as well an any new breeds using the afore mentioned breeds in out crossing or development programs.

## Background

During maturation of mammalian erythrocytes, the nucleus and cellular organelles are lost [1,2]. Thus, the mature erythrocytes lack DNA and are unable to produce RNA, rendering them incapable of division and repair and limiting the functional lifespan of the circulating red blood cell (RBC). Additionally, mitochondrial loss limits ATP production within the RBC to glycolysis. The final ATP yielding step of the Embden-Meyerof glycolytic pathway is the conversion of phosphoenolpyruvate to pyruvate via the regulatory enzyme pyruvate kinase.

Two structural genes encode pyruvate kinase (PK); *pyruvate kinase, liver and RBC* (*PKLR)* and *pyruvate kinase, muscle* (*PKM2)*[3]. Tissue- and development-specific expression, as well as cellular metabolic needs, determine which of these genes is expressed [4-8]. Additionally, each gene has isozymes generated by alternative splicing of exons 9 and 10 in *PKM2* and promoter selection, with the corresponding exon 1, in *PKLR* L and R alleles [9,10]. *PKM2* generates the M1 and M2 isozymes. M1-PK is produced in muscle and brain while M2-PK is expressed in retina, lung, fat tissue, pancreas and rapidly dividing cells such as embryonic and tumor cells [11]. In humans, the L and R isozymes are *PKLR* products generated in the liver and reticulocytes, respectively. Homologous sequence data protein prediction algorithms suggest both forms are present in other primates but experimental evidence for two forms is only supported in humans, dogs (NM_001256262.1 and NM001256018.1), mice (NM_013631.2, NM_001099779.1) and rats [12].

Perturbation of PK results in PK deficiency and ultimately leads to hemolytic anemia. In humans, over 190 PK mutations have been identified [13-21]. PK deficiency has also been clinically characterized in several canine small-breed dogs including: Basenji [22], Beagle [23], Dachshound [24], Cairn Terrier [25], Pug [26], and West Highland White Terrier [27]. To date, affected large breed dogs have been restricted to Labrador Retrievers [26]. An exonic deletion (c.433delC) results in PK deficiency in Basenjis [28], a 6 bp exon 10 insertion is causative in West Highland and Cairn Terriers [26,29], missense mutations c.848T>C and c.994G>A result in Pug and Beagle PK deficiency respectively and Labrador Retrievers have a c.799C>T mutation that results in a premature termination codon and loss of over 53% of the protein [26]. It should be noted that not all Labradors with clinical symptoms of PK deficiency have the c.799C>T mutation [26]. Affected dogs present with clinical signs as early as a few months to as late as several years of age regardless of the causative mutation. Disease severity is variable but both Basenjis and Labradors with the c.799C>T mutation have a more severe anemia and shortened lifespan [26]. The initial sign of the disease commonly presents as exercise intolerance resulting from PK deficiency induced anemia.

Domestic cats also have an inherited PK deficiency. The disease manifests as a chronic, intermittent, hemolytic anemia. Typically, clinical symptoms include but are not limited to: lethargy, diarrhea, pale mucous membranes, lack of appetite, poor coat quality, weight loss, icterus and occasionally splenomegaly. Blood chemistry may reveal anemia, increased aggregated reticulocyte counts, hyperglobulinaemia, hyperbilirubinaemia, and increased liver enzymes (for detailed, longitudinal clinical symptoms see Kohn et al. 2000 and 2008) [30,31]. In a cohort of 25 affected cats, clinical signs first manifested as early as six months and as late as five years. Some cats died or required euthanization while others maintained an adequate quality of life. Thus, both severity of clinical presentation and age of onset are variable.

The first documented report of feline PK deficiency was in an Abyssinian cat [32]. Later, reports demonstrated that Somalis, a longhaired variety of Abyssinians, as well as random bred domestic shorthairs, had PK deficiency. A proceedings abstract implicated a deletion of the last 13 bases of exon 5 of R type PKLR from liver cDNA in Abyssinian and Somali cats [33]. No correlating genomic deletion was detected but two intron 5 SNPs were mentioned, one of which was concordant with disease. The nature, sequence, and location of the SNPs were not disclosed. No sequence, gene, or SNP data has ever been curated in any publicly available repository regarding PK deficiency. However, subsequent research evaluating geographic distribution and frequency of the PK deficiency SNP in the Abyssinian and Somali breeds, performed in collaboration with the reporting laboratory, cites the proceedings abstract [30,34].

In this study, *PKLR* mRNA was isolated from whole blood and bone marrow from two cats heterozygous for the historically proposed PK mutation and the generated cDNA was examined in its entirety for additional mutations by sequence analysis. A concordant SNP at position 304 of intron 5 was identified, as well as two, non-correlated SNPs at intronic positions 303 and 320. The disease-associated SNP presence and frequency was determined in 14,179 cats representing 40 breeds or populations. Fifteen groups (defined as a specific breed, samples that could not be assigned to a breed and domestic random bred cats) possess the diagnostic PK deficiency SNP at a frequency of 9.35%.

## Methods

### Samples for population frequencies

Buccal samples from cats (n = 14,179) were submitted to the Veterinary Genetics Laboratory (VGL) (n = 12,630) at the University of California at Davis, CA or Langford Veterinary Services (LVS) (n = 1,549) at the University of Bristol, UK for genetic testing. Breed identification was owner reported. DNA from VGL submitted samples was isolated as previously described [35]. LVS submitted samples were supplied from owners as buccal swabs and DNA isolated as described previously [36].

### RNA and DNA isolation

Genomic DNA was isolated from peripheral RBC from two cats heterozygous for the putative diagnostic SNP and from a cat homozygous for the SNP but not presenting with clinical signs of anemia. Total RNA was isolated from bone marrow and liver samples from one carrier cat and from RBC of the second carrier cat. Tissues were stored in RNAlater (Qiagen Inc., Valencia CA, USA) at −20°C prior to RNA isolation. All RNA was isolated as previously described [37]. Samples were collected from research animals housed at the Feline Genetics Research Colony at UC Davis under Institutional Animal Care and Use Committee protocol 15177.

### Gene analysis

Feline genomic sequence data for *PKLR* generated by the feline genome sequencing project (AANG00000000.2) [38] was available for exons 2 and 6 through 11. Primers were designed using Primer3plus ( http://www.bioinformatics.nl/cgi-bin/primer3plus/primer3plus.cgi) flanking the exons to obtain splice boundaries (Table 1). No genomic sequence was available in the public database for cat *PKLR* exons 1, 3, 4, and 5. Complete complementary cDNA was generated using Superscript III first strand synthesis reaction (Invitrogen, Carlsbad, CA) according to manufacturer’s gene specific primer protocol. 

**Table 1:** **Genomic and cDNA primers and their locations for*PKLR*in domestic cats**

**gDNA Primer**	**5'-3' Sequence**	**gDNA Primer**	**5'-3' Sequence**
PKLRex2F	CGGTACTTGCTCCACAGGAT (chrF1:88325815–34)*	PKLRex2R	GTGGCGATGATGCAGGTACT (chrF1:88326141–60)
PKLRex5F	CGAACACCGTGTGGGTAGACTA (derived from cDNA)	PKLRex6R	GGAGGCAAACACGATGTCTACC (chrF1:88327517–38)
PKLRex6F	TCGTGTTTGCCTCCTTTG (chrF1:88327525–42)	PKLRex10R	AGACCTCTCGGAACAGGTG (chrF1:88329412–30)
PKLRex9F	TCAAGTGCTGTGCTGCTG (chrF1:88328580–97)	PKLR3utrR	TGGCCAGTGCTTAATTGG (chrF1:88330288–305)
**cDNA Primer**	**5'-3' Sequence**	**cDNA Primer**	**5'-3' Sequence**
PKLR_cD_Ex2F	GGGTACCTACGGCGGGTCAG (chrF1:88325991–6010)	PKLR_cD_Ex7R	CTTCTCAGGTGGGATCTCGAT (chrF1:88327825–45)
PKLR_cD_Ex6F	TCGTGTTTGCCTCCTTTGTGCG (chrF1:88327525–42)	PKLR_cD_Ex10R	AGACCTCTCGGAACAGGTG (chrF1:88329412–30)
PKLR_cD_Ex8F	GAGACGAGCGATGTAGCG (chrF1:88328256–73)	PKLR_3UTR	CGTGAAATGGAGCAGGGAAGG (chrF1:88330247–67)
**5' Race primer**	**5'-3' Sequence**	**5' Race primer**	**5'-3' Sequence**
AUAP	GGCCACGCGTCGACTAGTAC	PKLR_5'_ex2/3R	CCGGCCCAATGGTGGCG

* locations defined by feline assembly Dec. 2008 NHGRI/GTB V17e/felCat4.

Genomic DNA was amplified for available exons as previously described [39]. All cDNA PCRs were performed as two separate reactions in a total volume of 20 μl. First round PCRs used 2 μl cDNA as the template while second round reactions used 1 μl of the first round amplicons in the following reaction: 2 mM MgCl_2_, 1X Amplitaq Gold Buffer (Applied Biosystems), 1.25 mM dNTPs, 0.5 μM of each primer and 0.75 units of Amplitaq Gold (Applied Biosystems). First round PCR reactions were amplified under the following cycling conditions in a 2720 Thermal cycler (Applied Biosystems): 94°C for 4 min initial denaturation, followed by 5 cycles of 30 sec at 94°C, 2 min at 72^o^ C, then 5 cycles of 30 sec at 94°C, 2 min at 70°C and lastly 25 cycles of 30 sec at 94°C, 20 sec at 66°C and 2 min at 72°C. A 72°C final incubation for 10 min was added to ensure full product length for all amplicons. The second round thermal cycle profile was as follows: 94°C for 4 min initial denaturation, followed by 5 cycles of 30 sec at 94°C, 20 sec at 64°C and 2 min at 72°C, 5 cycles of 30 sec at 94°C, 20 sec at 62°C and 2 min at 72°C and 25 cycles of 30 sec at 94°C, 20 sec at 60°C and 2 min at 72°C, with a final extension at 72°C for 10 min. PCR products were visualized by agarose gel electrophoresis and prepared and sequenced as previously described [40]. Sequencing products for an individual cat were assembled into a single gene contig using Sequencher analysis software v4.1 (Gene Codes Corporation, Ann Arbor, MI). Assembled contigs from each sample were aligned to identify sequence variants.

The 5' untranslated region and exon 1 sequence data were obtained using the GeneRacer Core Kit (Invitrogen) as previously described [37]. Amplified products were sequenced and assembled as described above.

## Results

The complete PK type-R transcript sequence was obtained from both a bone marrow and liver sample of a carrier cat. The feline CDS R-type PK transcript is 1,725 bp, contains 11 exons, and *in silico* translation predicts a 574 amino acid protein. Sequence identity to domestic dog, panda and human (GenBank Accession Nos.: NM_001256262.1, XM_002928283, NM_000298, respectively) is 89.9%, 89.7% and 87.6%, respectively, with a protein homology of 89.7%, 88.7% and 88.0%, respectively. A partial PK transcript was obtained from RBC, but because it lacked the 5’ portion it could not be identified as an R-type or L-type transcript. Multiple attempts failed to obtain the L-type mRNA transcript from liver, bone marrow and RBCs.

**Figure 1:**
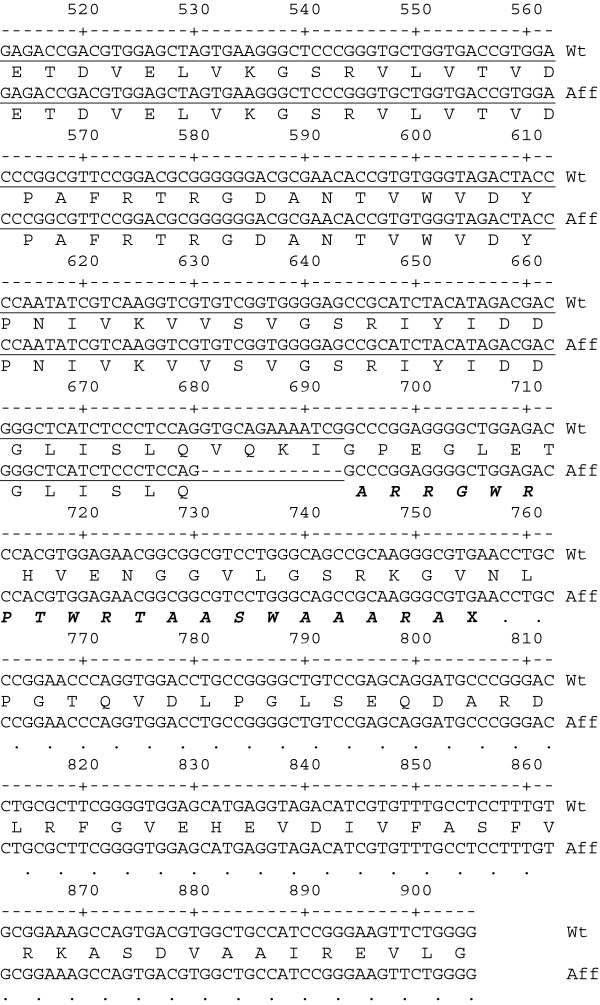
**cDNA sequence and protein alignment of *PKLR *exons 5 and 6 for the domestic cat.** Wild type (Wt) and affected (Aff) exon 5 (underlined) and 6 cDNA sequence data are shown. Base pair numbering is from the *PKLR* translation start. Protein translation is shown below each cDNA sequence. Altered affected PKLR protein is italicized in bold. A dot in the figure represents no predicted protein translation product. Standard IUPAC nomenclature is followed.

Bone marrow and liver RNA from the carrier cat yielded the wild-type-R transcript as well as a shortened product (GenBank Accession #s JX951426 and JX951427). The abbreviated transcript is identical to wild-type at the sequence level with the exception of a deletion of the last 13 bp of exon 5 (Figure 1). The same abbreviated transcript was identified in the RBC of a second carrier cat, however, the 5’ portion was not obtained. *In silico* translation of this product results in a frame shift and erroneous translation of the first 20 amino acids of exon 6, followed by a premature stop codon at position 248 (Figure 1). This truncation results in a loss of the terminal 57% of the mature *PKLR* type-R protein.

**Figure 2:**
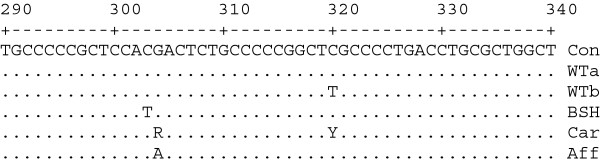
**Partial genomic DNA intron 5 sequence of *PKLR *in the domestic cat.** Base pair positions are numbered from the exon 5 splice boundary. The correlated PK deficiency SNP is found at position 304. Three non-affected cats (WTa, WTb, and BSH-British shorthair) are shown as well as a carrier (Car) and affected (Aff). A dot represents positions where the sequence data matched the feline consensus (Con) sequence. Standard IUPAC nomenclature is followed.

Genomic sequencing of two wild-type, one carrier and the cat homozygous for the putative causative SNP for *PKLR* exon 5, intron 5 and exon 6 revealed two SNPs in intron 5 at positions 304 and 320; a G>A and C>T, respectively (Figure 2) (GenBank accession # JX951425). No other polymorphisms were identified in this region or in the other genomic sequence evaluated that included exons 2, 6 – 11, their intron-exon boundaries and portions of the flanking intronic regions. The c.693+304G>A mutation was concordant with the cat PK deficiency phenotype, while the c.693+320C>T mutation was homozygous in cats with no clinical history of anemia and therefore presumed discordant. While typing submitted samples, LVS noted that British Shorthairs contain a c.693+303C>T mutation on the wild type allele that is discordant with the disease phenotype.

Cat breed samples were submitted by owners to the VGL and LVS for PK deficiency testing, which evaluated the *PKLR* c.693+304G>A SNP. The cats submitted for DNA testing represented a biased and unbiased sampling for estimation of the SNP frequency (Table 1). Biased samples included those specifically submitted for PK deficiency testing (n = 3,084 - VGL, n = 1,506 - LVS) and the unbiased samples were submitted for other cat-specific DNA tests (n = 9,546 – VGL, n=43-LVS). Thirty-seven breeds were submitted to the VGL and six to LVS, representing 38 cat breeds from USA and UK populations. Cat samples submitted to the VGL were primarily from United States sources but also included international submissions.

At least 10 unrelated individual cats were required to represent a population in this study. The c.693+304G>A PK deficiency-associated SNP is present at a frequency of 6.31% for the entire sample set (Table 2). The c.693+304G>A SNP was detected in 13 breeds, random bred cats and unspecified cats and was absent in 25 breeds. The frequency of the c.693+304G>A increases to 9.35% when considering only these 15 groups that had at least one presentation of the SNP. The frequency of the c.693+304G>A SNP in unbiased samples from breeds known to carry the SNP ranged from 0.078% in the Exotic Shorthair to 12.97% in the Bengal (Table 3). In submitted (biased) samples requesting PK deficiency testing (Table 4), the SNP frequency ranged from 0.0% in the Australian Mist cats to 41.4% in the Singapura breed from the UK. United States PK deficiency test requesting samples ranged from 11.75% in the Somali to 23.0% in the Singapuras.

**Table 2:** Pyruvate kinase deficiency allele frequencies in cat breeds and populations

**Breed**	**Samples**	**GG**	**GA**	**AA**	**Freq. A**	**Exp GG**	**Exp GA**	**Exp AA**
Abyssinian*	1955	1476	465	14	0.126	1493	431	31
Abyssinian (UK)	36	28	8	0	0.11	29	7	0
American Bobtail	10	10			0			
American SH	51	51			0			
American Wirehair	11	11			0			
Australian Mist (UK)	27	27			0			
Balinese	39	39			0			
Bengal*	1340	925	390	25	0.164	936	368	36
Bengal (UK)	1210	827	354	29	0.17	834	341	35
Birman	187	187			0			
British SH	966	966			0			
British SH (UK)	43	43			0			
Burmese	118	118			0			
Burmilla	38	38			0			
Cornish Rex	46	46			0			
Devon Rex	186	186			0			
Domestic SH/LH	76	62	13	1	0.099	62	13	1
Egyptian Mau	36	27	9	0	0.1	28	8	1
Exotic LH	91	91			0			
Exotic SH	645	644	1	0	0.0008	644	1	0
Himalayan	601	601			0			
Korat	41	41			0			
La Perm	56	40	16	0	0.14	41	14	1
Maine Coon	118	103	15	0	0.064	104	14	0
Munchkin	19	19			0			
Norwegian Forest Cat	13	10	3	0	0.1	10	3	0
Ocicat	69	69			0			
Oriental LH	48	48			0			
Oriental SH	337	336	1	0	0.001	336	1	0
Persian	2226	2217	9	0	0.002	2217	9	0
Ragamuffin	20	20			0			
Ragdoll	1063	1063			0			
Russian Blue	14	14			0			
Savannah Cat	11	10	1	0	0.05	10	1	0
Scottish Fold	68	68						
Selkirk Rex	124	124						
Siamese	457	457						
Siberian Cat	377	360	16	1	0.024	359	18	0
Singapura	150	88	55	7	0.23	89	53	8
Singapura (UK)	168	60	77	31	0.414	58	81	29
Somali	633	527	104	2	0.0853	530	99	5
Somali (UK)	65	49	14	2	0.14	48	16	1
Sphynx	248	248						
Turkish Angora	31	31						
Unreported	111	98	13	0	0.059	99	12	0
**Total**	**14179**	**12503**	**1564**	**112**	**0.063051**	**12447**	**1675**	**56**

*=breeds significantly deviating for Hardy-Weinberg expected ratios.

**Table 3:** Unbiased PK deficiency allele frequencies in 12 cat breeds based on samples submitted for other genetic testing

**Non - PK deficiency submitted samples (unbiased)**								
**Breed**	**#**	**GG**	**GA**	**AA**	**Freq. A**	**Exp GG**	**Exp GA**	**Exp AA**
Abyssinian	497	445	52	0	0.0523	447	49	1
Bengal	320	242	73	5	0.130	242	72	5
Domestic SH/LH	76	62	13	1	0.12	59	16	1
Egyptian Mau	36	27	9	0	0.13	27	8	1
Maine Coon	118	103	15	0	0.0636	103	14	0
Exotic SH	645	644	1	0	0.000775	644	1	0
Norwegian Forest Cat	13	10	3	0	0.12	10	3	0
Oriental SH	337	336	1	0	0.00148	336	1	0
Persian	2226	2217	9	0	0.002021	2217	9	0
Savannah	11	10	1	0	0.046	10	1	0
Siberian	377	360	16	1	0.0239	359	18	0
Somali	227	213	14	0	0.0308	213	14	0
unreported	111	98	13	0	0.0586	98	12	0
**Total**	**4994**	**4767**	**220**	**7**	**0.0234**	**4763**	**229**	**3**
**Total** (−Per, OSh, ExSh)	**1786**	**1570**	**209**	**7**	**0.06234**	**1570**	**209**	**7**

**Table 4:** Biased PK deficiency allele frequencies in six cat breeds based on samples submitted for PK testing

**Samples submitted for PK deficiency testing (biased)**								
**Breed**	**#**	**GG**	**GA**	**AA**	**Freq. A**	**Exp GG**	**Exp GA**	**Exp AA**
Abyssinian*	1458	1031	413	14	0.1512	1051	374	33
Abyssinian (UK)	36	28	8	0	0.11	29	7	0
Abyssinian Total	1494	1059	421	14	0.1492	1080	381	33
Aust Mist (UK)	27	27	0	0	0.0	27	0	0
Bengal*	1020	683	317	20	0.1750	694	295	31
Bengal (UK)	1210	827	354	29	0.1702	833	342	35
Bengal Total	2230	1510	671	39	0.1724	1527	637	66
La Perm	56	40	16	0	0.1429	41	14	1
Singapura	150	88	55	7	0.230	89	53	8
Singapura (UK)	168	60	77	31	0.414	58	81	29
Singapura Total	318	148	132	38	0.327	147	134	37
Somali	400	308	90	2	0.118	311	83	6
Somali (UK)	65	49	14	2	0.14	48	16	1
Somali Total	465	357	104	4	0.120	359	99	7
**Total US**	**3084**	**2150**	**891**	**43**	**0.1584**	**2186**	**819**	**79**
**Total UK**	**1506**	**991**	**453**	**62**	**0.1916**	**968**	**446**	**65**
**Total**	**4590**	**3141**	**1344**	**105**	**0.1693**	**3113**	**1251**	**143**

*=breeds significantly deviating from Hardy-Weinberg expected ratios.

For 13 of 15 groups with the PK deficiency associated SNP (Table 2) observed and expected genotypes based on the SNP frequency in the populations did not deviate significantly from expected Hardy -Weinberg ratios at p = 0.05. However, significant Hardy-Weinberg deviations were found for Bengals and Abyssinians at p = 0.05 and 0.001, respectively but only for the US populations. Observed carriers were increased in the Bengals by 6.0%, and homozygous affected individuals were decreased by 30.6%. In Abyssinians, carriers increased by 7.9% while the observed homozygous affected cats decreased by 54.8%. When considering the biased versus unbiased Abyssinian and Bengal samples, the observed genotypes for the unbiased sample sets had Hardy-Weinberg predicted genotype ratios. However, the biased sample set significantly deviated at p = 0.001 for both data sets. The Abyssinians had a 10.4% increase in carrier cats and a 57.6% reduction in observed homozygous affected cats while Bengal cats had a 35.5% reduction in observed homozygous affected cats.

## Discussion

Glycolysis is one of the principal pathways of energy (ATP) generation in cells. In erythrocytes, glycolysis is the only pathway for ATP synthesis since mature red cells lack mitochondria. The most frequent glycolytic abnormality is PK deficiency. In humans, most mutations are located in the coding sequences of *PKLR*, with missense, deletions, insertions, splice defects, premature stop codons and promoter mutations, being common. Humans, mice, dogs and cats have been documented to have PK deficiency [22,23,25,27,29,32,41-43]. In cats, the disease was first reported in Abyssinians and their longhaired morph, the Somali, as well as in random bred cats [31,32]. Subsequent to the reports in Abyssinians, breeders and veterinarians have suggested that additional breeds demonstrate the hallmarks of Abyssinian PK deficiency by requesting the PK deficiency test from the service laboratories.

In a 2005 detailed review, Zanella et al. [20] determined that 158 novel human *PKLR* mutations had been identified. Subsequent population studies have increased this total by over 44 novel mutations [18,44]. Mutations are found in every exon, as well as several introns, all of which result in human PK deficiency. The domestic cat *PKLR* type-R CDS transcript has similar genetic sequence and structure to other mammalian species, with ~88% homology at both the DNA and protein sequence level. Between species polymorphisms are distributed across the transcript. This study demonstrated that cats with PK deficiency produce transcripts with a 13 bp deletion at the 3' end of exon 5 of *PKLR,* however the altered form was only confirmed in the R-type transcript found in liver, bone marrow and blood. The deletion results in the loss of 57% of the mature protein, likely resulting in the disease phenotype. The only identified and correlated genomic mutation is a guanine to adenine transition in intron 5, 304 bp 3' of the exon 5 splice-donor site and 53 bp upstream from the exon 6 splice-acceptor site. The mechanism by which this mutation results in the erroneous splicing of exons 5 and 6 has yet to be determined. Several splice site variants have been noted in human PK deficiency patients, and how these affect splicing has not always been identified [45,46]. For example, the effect of mutation IVS9 +43c on the mRNA splicing cannot be directly demonstrated because the aberrant mRNA transcript cannot be obtained. However, the IVS9 +43c mutation is likely to be involved in PK deficiency since the mutation is not found in normal human populations, *in silico* splicing prediction suggests introduction of a new acceptor splice site, and no other sequence abnormalities were detected in the patient's DNA [16]. Moreover, mutations in internal portions of exons or introns, which result in aberrant splicing, have been described in several disease-related genes [47,48].

PK activity in the erythrocytes of carrier cats is estimated as 50% the level of activity of the wild type [32] yet the frequency in the random population may suggest some selective advantage for cats with the mutation such as blood -borne parasite infection resistance. Homozygous cats that should have disease-associated presentations, such as splenomegaly, hemolytic anemia and increased osmotic fragility of erythrocytes, are not being actively reported by Bengal, Singapura and other breed owners to the veterinary community possibly owing to the fact that the disease can be episodic, mild and sub-clinical [30]. In addition, the fact that expression of the disease phenotype is variable both in the age of onset and severity suggests that additional factors may be required to induce disease, such as stress and activity level [30]. Never the less, the lack of presentation of sick cats is puzzling, prompting further investigation of the c.693+304G>A PK deficiency-associated SNP.

PK deficiency was first documented in an Abyssinian from the USA [32], and later in Abyssinian and Somali cats in Germany and Australia [49,50]. A limited Australian population study in Abyssinian and Somali cats (N = 60) revealed a frequency of 0.13 and 0.29, respectively, of the intronic SNP [34]. However, the authors noted that, although multiple breeders were approached for inclusion, participation may have been biased towards those with known carrier cats. Additionally, related cats, including full siblings, were used. Thus, the estimation of prevalence in Australia may be significantly biased towards the affected allele. Abyssinian and Bengal breeders have been actively screening for the disease-associated SNP and represent over 81% of samples submitted for PK deficiency testing. The c.693+304G>A PK deficiency-associated SNP allele frequency deviates from Hardy-Weinberg equilibrium (p=0.05) (Table 2) only in these two breeds. The Abyssinians, which have been tested for PK deficiency over the longest interval, have a significant deficit from the predicted numbers of homozygous affected cats. The Bengals also have a decrease in the observed numbers of homozygous affected cats, although to a lesser extent. Increases in the observed number of carriers in both breeds would suggest that breeders are using genetic data to actively select against matings that would produce affected offspring. Moreover, lack of selection against the deleterious allele in the remaining populations has allowed the c.693+304G>A PK deficiency-associated SNP to persist and results in higher percentages of affected cats in those breeds.

In this study, the frequency of the c.693+304G>A PK deficiency-associated SNP ranged from 3 – 15% in Abyssinian and Somalis between the USA and UK. Although Abyssinian and Somali breeders have known of the health concern, no genetic test has been peer-reviewed and published to ensure widespread detection of the mutation. Consequently, in an unbiased sampling of cats, the frequency of the c.693+304G>A PK deficiency-associated SNP is ~13% in Bengals and Egyptian Maus, two breeds that have used Abyssinians in their breed development, and perhaps would have benefitted from better knowledge of the potential of PK deficiency concerns. The c.693+304G>A *PKLR* SNP is at a critically high level in the Singapura with nearly 42% of UK tested cats having the SNP. This sampling is likely biased as breeders with concerns for the disease are submitting cat samples for testing. However, the frequency in Bengals in the UK (16%) is similar to the USA (18%) and nearly the same for unbiased Bengal sampling in the USA (13%). The Singapura is also a breed developed from Abyssinian stock [51]. Thus, the introgression of the disease into so many distinct breeds is not enigmatic. In Singapuras, the SNP frequency (32.7%) is 2.6-fold greater than that of the remainder of the affected breeds (12.47%). The Singapura is numerically one of the smallest breeds and the high frequency likely results from either a probable high frequency of the c.693+304G>A *PKLR* SNP in the founder population or accidental high inbreeding of affected cats during the breeds inception in the 1970's. Lack of a diagnostic test in the early stages of breed development and later, failure to disclose the genetic marker correlated with the disease undeniably contributed to the spread of the affected allele via breed development programs.

The western breeds, Norwegian Forest cats, Siberians and Maine Coons, are derived from regional feral populations in Scandinavia, Russia and the USA, respectively. Considering the most probable explanation of identity by decent, the presence of the c.693+304G>A *PKLR* SNP in these three breeds presents an interesting conundrum regarding its origin. The SNP is present in random bred cats (11% in 76 cats). It is possible that random bred founder cats or local feral cats used in breed expansion carried the allele. It is equally likely that a desirable phenotype from an affected breed was introduced into these populations and the affected allele was inadvertently introduced as well.

The c.693+304G>A *PKLR* SNP allele frequency varied across the populations evaluated. Samples representing 25 groups did not possess the SNP. Breeds such as the Persians, Exotic and Oriental shorthairs had frequencies less than 0.002, a 10-fold lower frequency than found in the next lowest breed, the Siberian. The nine carrier Persian samples were provided by breeders from Japan (3), Chile (2), England (3) and the Netherlands (1) and each set contained full siblings, Netherlands excepted. Intentional or inadvertent incorrect breed designation by owners on submission forms may account for the presence of c.693+304G>A *PKLR* SNP in some breeds. Some breeders have acknowledged providing incorrect breed identification when submitting samples for testing to circumvent commercial laboratory rules limiting availability of tests that have not been validated for a specific breed. Thus, the breeds with extremely low frequencies may not actually have affected alleles within their populations.

The entire *PKLR* type-R transcript has now been sequenced. The lone transcript variant observed between affected and wild-type alleles is a 13 bp deletion at the 3' end of exon 5. This mutation is correlated with an intronic c.693+304G>A mutation. No other potentially causative mutation was identified although two additional intron 5 mutations are in close proximity to the causative mutation. Of particular interest is the British Shorthair c.693+303C>T mutation, located 1bp from the correlated mutation (Figure 2), which is present at a frequency of 27.9% but has no disease association. Thus, both the nature and location of the c.693+304G>A may be critical for disease presentation.

## Conclusions

This study presents an extensive survey for the presence and frequency of the c.693+304G>A PK deficiency-associated SNP in 38 domestic cat breeds with 12 breeds ranging from 2 - 16% for the SNP. Based upon allelic frequency in random unbiased population sampling, PK testing is recommended for several breeds including Bengals, Egyptian Maus, La Perms, Maine Coon cats, Norwegian Forest cats, Savannahs, Siberians, and Singapuras, in addition to Abyssinians and Somalis. Breeds known to have been derived from Abyssinian crosses such as the Ocicat and novel breed development or out-crossing programs using Bengals or other affected breeds should test as well. Future expanded testing will undeniably help prevent breeding affected individuals and possibly allow selective elimination of the c.693+304G>A PK deficiency-associated SNP in domestic cat populations.

## Abbreviations

PK: Protein Kinase; *PKLR*: Pyruvate Kinase, Liver and Red Blood Cell; SNP: Single Nucleotide Polymorphism; ATP: Adenosine Triphosphate; *PKM2* Pyruvate Kinase: Muscle; dNTP: Deoxynucleotide Triphosphate; cDNA: Complementary Deoxyribonucleic Acid; RNA: Ribonucleic Acid; mRNA: Messenger Ribonucleic Acid; RBC: Red Blood Cell; VGL: Veterinary Genetics Laboratory; LVS: Langford Veterinary Services; CA: California; UK: United Kingdom; USA: United States of America.

## Competing interests

The authors declare that they have no competing interests.

## Authors’ contributions

RAG performed the assays, sequenced the gene and cDNA and wrote the manuscript; JCG contributed to technical aspects of the manuscript and provided initial cross-breed data; MCTP and CRH coordinated sample assessment from service laboratories and edited the manuscript; LAL edited the manuscript. All authors read and approved the final manuscript.
